# Characteristics of Lipo-Oligosaccharide Loci of *Campylobacter jejuni* Isolates Associated with Guillain-Barré Syndrome from Hebei, China

**DOI:** 10.3390/ijms11031155

**Published:** 2010-03-19

**Authors:** Hai Jiang, Mao-Jun Zhang, Rui-Chun Liu, Xin-Ying Tian, Yi-Xin Gu, Jian-Zhong Zhang

**Affiliations:** 1 National Institute for Communicable Disease Control and Prevention, Chinese Center for Disease Control and Prevention, P.O. Box 5, Changping, Beijing 102206, China; E-Mails: jianghai@icdc.cn (H.J.); zhangmaojun@icdc.cn (M.-J.Z.); guyixin@icdc.cn (Y.-X.G.); 2 Department of Neurology, The Second Hospital of Hebei Medical University, Shijiazhuang 050051, China; E-Mails: liuruichun@yahoo.com.cn (R.-C.L.); tianxinying@yahoo.com.cn(X.-Y.T.)

**Keywords:** *Campylobacter jejuni*, Guillain-Barré syndrome, lipo-oligosaccharides

## Abstract

Ganglioside mimicry by *C.jejuni* lipo-oligosaccharides (LOS) could induce the production of autoantibodies against gangliosides and the development of Guillain-Barré syndrome (GBS). The LOS biosynthesis region exhibits significant variation with different strains. Using PCR amplifications of genes from published LOS loci and sequencing the LOS biosynthesis loci, the eight GBS-associated *C. jejuni* strains from HeBei could be classified into four classes. The expression of sialylated LOS structures (class A) or non-sialylated LOS structures(class F, H and P) in the *C. jejuni* LOS is considered to be two different factors for the induction of GBS.

## 1. Introduction

*Campylobacter jejuni* (*C. jejuni*) is the leading cause of acute bacterial gastroenteritis worldwide. It has been associated with the development of Guillain-Barré syndrome (GBS), a post-infectious polyneuropathy [1–4]. The association between *C. jejuni* and the development of GBS is thought to result from the molecular mimicry between outer core structures of bacterial lipo-oligosaccharides (LOS) and gangliosides in peripheral nerves [1,2,4–6]. LOS of *C. jejuni* display considerable variation in the outer core structure. Microarray and PCR probing studies have shown that there is extensive variation in the gene content of the locus [7]. Based on gene content and organization, the loci are divided into eight classes (Class A through H) [8]. Many studies indicate that the Class A locus is predominately associated with GBS [8–10]. A recent study identified 11 new classes of LOS loci that were distinctly different from the previous eight classes [11]. They demonstrated the insertions and deletions of genes were related among different LOS classes. In the present study, the characteristics of loci involved in the biosynthesis of the LOS outer core from eight *C. jejuni* strains isolated from GBS patients in Hebei, China were studied.

## 2. Results and Discussion

### 2.1. Gene Contents and Organizations of the LOS Biosynthesis Loci in 8 *C.jejuin* Strains

According to the general organization of the LOS biosynthesis genes, the eight *C. jejuni* strains in this study could be classified into four classes “A,” “F,” “H,” and “P”. Four strains, QYT, LXC, ZHX, and ZB, which have 13 ORFs and only one copy of *cgtA* gene (*orf5*) in a 12.2-kb locus, belonged to class A. The 12.2-kb DNA sequences among the four Class A strains had 98% identity. An alignment of the deduced protein sequences of the *cstII* gene (sialytransferase) from four class A strains gave 96% identity. Three strains (LXC, ZHX and ZB) had mono-functional CstII (Thr51) and strain QYT had bi-functional CstII character with Asn51, Leu-54, and Ile-269. There was also a missing A base at position 1,234 of *orf3* in these class A strains which were consisted with previous description [9].

Strain XWM has 9 ORFs in a 8.5-kb locus, and belonged to Class F. It lacks the *neuBCA* and *cst* genes and likely directs the synthesis of nonsialylated LOS structures. The DNA sequence of the 8.5-kb LOS of strain XWM showed a 99.7% identity with strain RM1170, and showed 99.3% identity with strain GB15, which were both identified as Class F [11].

Strain LC has 18 ORFs in a 14.8-kb locus, which showed a 99.0% DNA sequence identity to strain RM1047, belonged to Class H. Two strains, LL and ZX, belonged to Class P, which had 19 ORFs in a 15.8-kb locus with 99.8% DNA sequence identity to strain GB4 [8,11].

### 2.2. Discussion

So far, 46 ORFs were found in LOS loci and 19 major LOS classes: A-S, were identified based on gene content and organization of the LOS biosynthesis loci [1]. Classes A, B, and C were involved in sialylated LOS synthesis [9,12–16]. The present study described the LOS characteristics of eight *C. jejuni* strains isolated from GBS patients from north China. Four of them belonged to Class A, which was consistent with previous reports [8–10,16]. Classes F, H, and P were found in four strains that possessed non-sialylated LOS structure. The presence of these four classes of LOS loci demonstrated genetic rearrangements and the complexity of LOS biosynthesis loci in GBS associated *C. jejuni* strains. However, no additional experiments have been done on analysing the LOS structure and the gangliosides mimics of each strain. With respect to other non-ganglioside-like structures (class F, H and P) that may be involved in molecular mimicry in the development of GBS. Godschalk *et al*. have demonstrated that co-infection with multiple *C. jejuni* strains also occurs in GBS patients [10]. Co-infections with multiple *C. jejuni* strains might be an alternative explanation to these classes. Although the presence of certain *C. jejuni* genes involved in sialylated LOS biosynthesis may be crucial for induction of the anti-ganglioside immune response that leads to GBS, the genetic susceptibility of host may also contribute to differences in clinical outcomes of the patients [8,14,20].

Besides the gene content, DNA sequence of this locus from multiple strains indicated that *C.jejuni* used other mechanisms to vary its outer core: phase variation because of homopolymeric tracts, gene inactivation by the deletion of a single base (without phase variation) and single or multiple mutations leading to allelic glycosyltransferases [9]. Nam Shin *et al*. have observed that the absence of a β-1,2-glucosyl residue on Hep-II makes possible the sialylation of the inner β-1,3-Gal residue by CstII [18]. Gilbert *et al*. suggested that the inactivation of the second domain of *orf3* by a frameshift mutation is the genetic basis for the absence of a β-1,2-glucosyl residue on Hep-II [9]. In this study, we also found a missing A base at position 1,234 in *orf3* among 4 class A strains (QYT, ZB, ZHX, and LX), which might infer the inactivation of the second domain of Orf3 due to frameshift mutation [9]. One of the variable residues among the CstII versions can result in either a mono-functional CstII (Thr51) or a bi-functional CstII (Asn51), so it has been suggested that the expression of different ganglioside-like structures might have an impact on the outcome of the GBS [19]. The alignment of the Cst-II protein sequence verified that only a class A strain (QYT) possessed the residue Asn51. Phase variation using homopolymeric G tracts was responsible for some of the variations in LOS outer core structures [9,11]. It still needs to be determined whether *orf23* and *orf25* in the class H and P loci might exhibit different enzymatic specificities until the LOS structures for all of these strains are determined.

**Figure 1.**
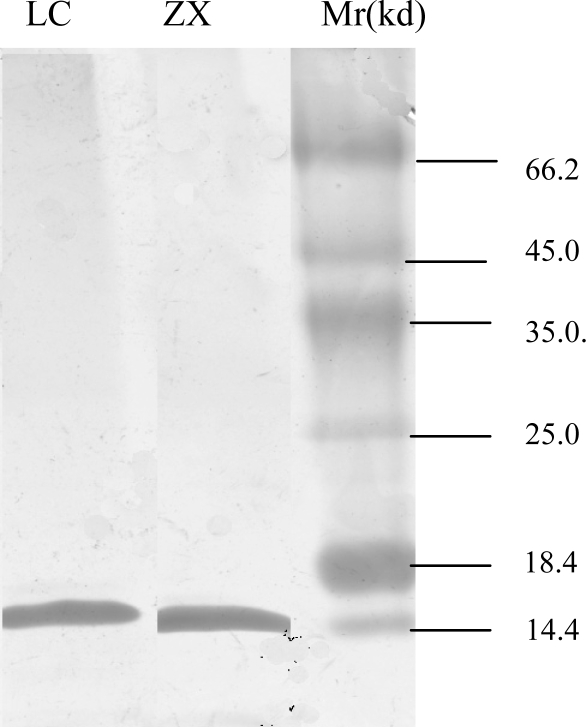
SDS-gels followed by silver staining of LOS extracts from *C. jejuni* LC (class H) and ZX (class P).

In this study, we found classes H (strain LC) and P (strain LL and ZX) shared similar gene content and organization, besides the deletion–insertion event of *orf28* and *orf39* with Classes E and H. The *orf28* gene was clearly missing in the Class H strain (LC) where *orf26* was disrupted by *orf39.* Indeed, the deletion–insertion event of *orf28* between class H and class P resulted in LOS differences, which led to a truncated LOS, as observed by SDS-PAGE (Figure 1). Based on BLAST searches, *orf28* was proposed to encode a putative glycosyltransferase and *orf39* was proposed to encode a putative butyryltransferase that was involved in the synthesis of the O-antigen of *E. coli* O91 [17].

Parker *et al* demonstrated that most of the GBS-associated strains (12 of 16 GBS strains) were HS:19 or HS:41, and 15 of them belonged to Class A, one belonged to Class C[8]. We found that only *C. jejuni* ZB and LXC were HS:19 and belonged to Class A. This reflected that the Penner serotype is not determined by the LOS characters.

## 3. Materials and Methods

### 3.1. Bacteria Identification and Serotyping

The eight *C. jejuni* strains were isolated from the stool specimens of eight GBS patients in the Second Hospital of Hebei Medical University in 1993. The isolates were cultured on Columbia agar supplemented with 5% sheep blood under micro-aerophilic conditions (5% O_2_, 10%CO_2_, and 85% N_2_) at 42°C for 24 h. Bacteria colonies were characterized by Gram stain, oxidase, catalase tests, and API Campy identification system test (API, REF 20800). *Campylobacter* species-specific PCR for *C. jejuni, C. coli, C. lari, C. upsaliensis,* and *C. fetus* were performed according to previous reports. The identified *C. jejuni* isolates were serotyped by the heat-stable (HS) serotyping scheme of Penner and Hennessy using a commercial 25 Penner heat-stable antisera set (*Campylobacter* Antisera Seiken Set, Denka Seiken Co., Tokyo) previously described. The serotype results are listed in Table 1.

### 3.2. PCR and PCR Products Sequencing

Genomic DNA of each strain was extracted with the DNeasy tissue kit (QIAGEN, USA). For the different size of PCR products, six pairs of primers were used based on the sequences of *C. jejuni* NCTC 11168 and 81116 LOS loci. The primers in LOS loci are listed in Table 2. PCR products were purified by PCR gel extraction kit (QIAGEN) and cloned in vector pGEM-T. The inserts were sequenced by primer walking with an ABI Prism Big Dye Terminator (v3.1) cycle sequencing ready reaction kit (v5.0).

### 3.3. DNA Sequence Alignment and Assigned GenBank Accession Number

The whole DNA sequence of the LOS biosynthesis loci of eight GBS-associated *C. jejuni* strains were compared with 19 published classes loci (Class A-S) that belonged to ATCC43432(A), ATCC43449(B), NCTC11168(C), LIO87(D), 81116(E), RM1170(F), ATCC43437(G), ATCC43431(H), RM1850(I), RM1508(J), GB24(K), RM3435(L), RM1503(M), RM2095(N), RM3423(O), GB4(P), RM3437(Q), GC149(R), and RM3419(S), which GenBank accession numbers are AF215689, AF401529, AL139077, AF400669, AJ131360, AY434498, AY436358, AF411225, EU404107, EU404104, AY573817, EU404111, EF140720, AY816330, EF143352, AY943308, EU404112, AY962325, and EU404110, respectively.

The nucleotide sequences of LOS biosynthesis loci from eight GBS-associated *C. jejuni* strains were deposited in *GenBank* and were assigned with the accession numbers as follows: LLCj LOS locus, DQ535890; QYTCj LOS locus, DQ864651; ZXCj LOS locus, DQ535891; ZBCj LOS locus, DQ864653; ZHXCj LOS locus, DQ864652; LCCj LOS locus, DQ535892; LXCCj LOS locus, DQ864650; XWMCj LOS locus, EF176584.

## 4. Conclusions

In the present study, the LOS structure of four GBS associated *C. jejuni* strains from China were identified as class A. Three classes (classes F, H and P) without the genes required for expression of ganglioside mimicry were found in other four GBS associated strains. Co-infections with multiple *C. jejuni* strains occur in GBS patients might be an alternative explanation to these classes. Further LOS structure analysis would be helpful to explore the mechanism of *C. jejuni* associated GBS.

## Figures and Tables

**Table 1.** *Campylobacter jejuni* strains used in this study.

**Strain no.**	**Isolated Time**	**Isolated Place**	**Subtype (GBS)**	**Penner type (HS)**	**LOS locus class**
LLCj	1993	HeiBei Province	AMAN	37	P
ZXCj	1993	HeiBei Province	AMAN	37	P
LCCj	1993	HeiBei Province	AIDP	ND	H
LXCCj	1993	HeiBei Province	AMAN	19	A
QYTCj	1993	HeiBei Province	AMAN	2	A
ZBCj	1993	HeiBei Province	AMAN	19	A
ZHXCj	1993	HeiBei Province	AMAN	ND	A
XWMCj	1993	HeiBei Province	AMAN	ND	F

Notes: AMAN: acute motor axonal neuropathy; AIDP: acute inflammatory demyelinating polyneuropathy; ND: not determined.

**Table 2.** Primers used in this Study.

**Primer no.**	**Primer sequence5′-3′**
P1 a	AAAGAATACGAATTTGCTAAAGAGG
P2 b	ATCATAAAAATCACTTGCCAAAACT
P3 b	AATTTTCCAAGAGGAGCACATGCAC
P4 b	CTATGGTGTAAGTGGGCATTGGGCT
P5 b	AAAAGCCCAATGCCCACTTACAC
P6 a	TTGCCAAGGTGAAGTTTGAGTAA
P7 c	ACATATAGACCCCTGAGGTAATTCTTT
P8 c	GTTATTATTGCTGGAAATGGACCAAGT

a P1P6 used to amplify the LOS biosynthesis loci of *C. jejun*i XWM, which were located on *orf1* and *orf13* respectively, as reported previously [9].

b P1P2, P3P4 and P5P6 were used to amplify the LOS biosynthesis loci of *C. jejun*i LL, ZX and LC. P2P3 and P4P5 were located on *orf25* and *orf30* respectively from strain 81116.

c P1P7 and P8P6 were used to amplify the LOS biosynthesis loci of *C. jejuni* QYT, LX, ZB and ZHX. P7 and P8 were located on *orf7* from NCTC11168.
